# A metastable phase of shocked bulk single crystal copper: an atomistic simulation study

**DOI:** 10.1038/s41598-017-07809-1

**Published:** 2017-08-04

**Authors:** Anupam Neogi, Nilanjan Mitra

**Affiliations:** 1 Advanced Technology Development Centre, Indian Institute of Technology Kharagpur, Kharagpur, 721302 India; 2 Center for Theoretical Studies, Indian Institute of Technology Kharagpur, Kharagpur, 721302 India

## Abstract

Structural phase transformation in bulk single crystal Cu in different orientation under shock loading of different intensities has been investigated in this article. Atomistic simulations, such as, classical molecular dynamics using embedded atom method (EAM) interatomic potential and *ab*-*initio* based molecular dynamics simulations, have been carried out to demonstrate FCC-to-BCT phase transformation under shock loading of 〈100〉 oriented bulk single crystal copper. Simulated x-ray diffraction patterns have been utilized to confirm the structural phase transformation before shock-induced melting in Cu(100).

## Introduction

Copper is one of the most common elements worldwide. Because of the importance and widespread usage of the material, this element has been studied extensively (both experimental [see series of publications by Meyers and his group]^1, 2^ and numerical [see series of publications by Bringa, Germann, Ravelo and their groups]^3–8^) along with different alloys it forms. However, it is believed that Cu can only exist in face-centered-cubic (FCC) crystal structure based on which numerous phase diagrams have been prepared and used. In fact for many years, many experimental groups around the world use copper as the first target material in shock compression studies to develop and demonstrate new capabilities - loading conditions, diagnostics, analysis, and modeling - since Cu is believed to be a simple, well-studied material which remains FCC until melting^9^. Thereby, the discovery of an existence of body-centered phase of bulk Cu is expected to cause a revelation in the research community.

There is no work in existing literature which demonstrates the existence of body-centered phase for Cu in bulk without any constraint conditions. Under constraint conditions such as epitaxy, thin films of BCC Cu have been grown pseudomorphically on Pd{001}, Pt{001}, Fe{001} and Ag{001} substrate^10–13^. Phase transformation has also been reported using Neutron diffraction experiments on Cu-based shape memory alloys such as Cu-Zn-Al^14^, Cu-Al-Ni^15^, Cu-Al-Pd^16^, Cu-Al-Be^17^ in which Cu is constrained in an alloy form with other metals. It should be noted that discovery of new phase transformation of material in bulk form is not an ordinary event and requires a mix of experimentation, predictive computations and of course luck.

Several researchers have demonstrated through *ab* − *initio* studies that BCC phase of Cu is energetically unstable at ground state and at ambient temperature and pressure under tetragonal deformation^18, 19^ which eventually has resulted in controversy within the community of pseduomorphic epitaxy demonstrating presence of thin films of BCC Cu over different substrates. The controversy has finally been resolved through the explanation that stable substrates influence the phase change in Cu and it can happen only in thin films and not for bulk material or even thick films^20, 21^. Friedel^22^ postulated that even though BCC phase of Cu is energetically unstable at the ground state, it may be preferred to the system at high temperature due to its large entropy resulting from low-energy vibrational transverse modes. It has been demonstrated that structural transition may be only driven by the excess of vibrational entropy of the high-temperature phase^23^. In fact for Cu-based shape memory alloys, Planes *et al*.^24^ demonstrated that low energy of the TA2[110] phonon vibrational mode provides the significant contribution to the excess of entropy which stabilizes BCC phase at high temperature, which has eventually been proved experimentally through a series of calorimetric and magnetic measurements^25^. Within the context of Landau theory, Planes *et al*.^26^ provided experimental evidence that coupling between homogeneous shear and short wavelength phonon is also an essential mechanism to account for martensitic transformations of Cu-based alloys.

The previous studies set the stage for an investigation of the possibility of phase transition in Cu at high temperature and pressure conditions. Hirth *et al*.^27^ postulated analytically that shock loading of Cu along the 〈110〉 direction would lead to the formation of a body-centered orthorhombic (BCO) phase. Levitas and Ravelo^28^ observed loss of coordination number of the crystals behind the shock front for a shock loaded Cu in the 〈110〉 and 〈111〉 directions, which they proposed as “virtual melting”. The detection of phase transformation of bulk Cu under high pressure and temperature conditions (as observed in shock experiments) remained elusive. Within the context of a polycrystal, Bolesta and Fomin^29^ demonstrated nucleation of solid-solid phase transition in Cu behind a shock wave front. The effects of grain size, grain orientation of the polycrystals of Cu under shock loads of different intensities with regards to structural phase transition has also been investigated^30^. However, it should be noted that a polycrystal material is a summation of different single crystal materials of different grain sizes in different orientations having distinct grain boundaries between them. Thereby identification of body-centered phase does not properly identify which direction of single crystal of bulk Cu is more prone to phase transition. Moreover, it is also not known from the polycrystal study whether the defect induced due to grains is responsible for the phase transformation. These questions are being specifically targeted in this study with regards to the structural phase transition of Cu under shock loading.

## Results

In this work multi-million atom classical non-equilibrium molecular dynamics (NEMD) simulations (based on many-body embedded atom method (EAM) interatomic potential for Cu parameterized by Mishin *et al*.^31^) has been carried out for simulating piston driven shock compression of single crystal FCC copper with different orientations, 〈100〉, 〈110〉 and 〈111〉 for the piston velocity of 0.8–3.0 km/s. For the shocks propagating along 〈100〉 and 〈110〉 a structural phase transformation, FCC-to-body centered phase of single crystal copper has been identified, as characterized through adaptive-common neighbor analysis (a-CNA)^32^, radial distribution function (RDF) and x-ray diffraction (XRD) pattern analysis which has been discussed in details in this paper. Typically, the NEMD simulations are carried out for several pico-seconds timescale; thus in order to test the stability of the newly identified phase for longer time periods multi-scale shock technique (MSST)^33^ simulations have also been carried out using same EAM potential of Cu^31^ for smaller target samples, up to 10 ns timescale. Questions could be raised with regards to whether the observed phenomenon of structural phase transition is primarily due to the choice of the force potential^34–36^; thereby, *ab* − *initio* molecular dynamics (AIMD) simulations are also performed for the same range of piston velocities. Details of the different simulation methodologies utilized have been provided in the METHODS section.

**Figure 1:**
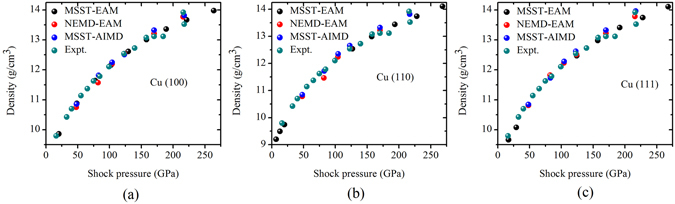
Shock Hugoniot point, pressure and density of the target sample with desired orientation (i.e. 〈100〉, 〈110〉 and 〈111〉) has been measured for all sets of simulations [MSST (utilizing EAM potential), NEMD (utilizing EAM potential) and *ab* − *initio* molecular dynamics (AIMD)] and compared with the available experimental shock Hugoniot data points^37^ for poly-crystalline Cu. The above figures represent shock data for different shock directions, (**a**) 〈100〉, (**b**) 〈110〉 and (**c**) 〈111〉.

**Figure 2:**
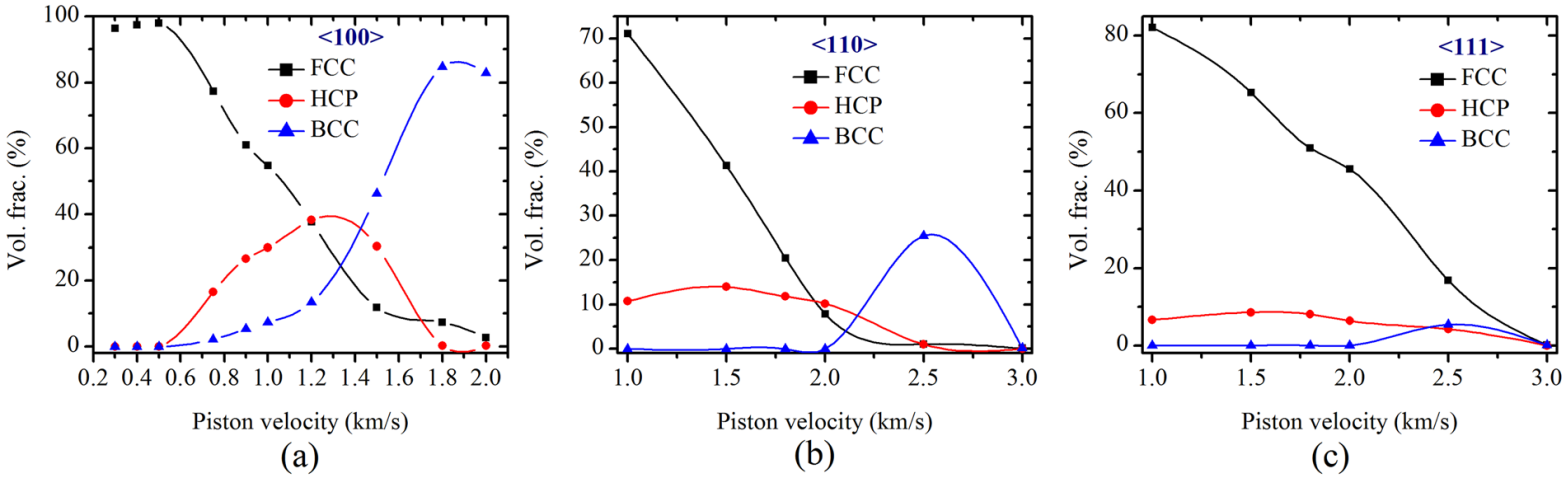
Evolution statistics of different nucleated phases like BCC, HCP from initial undeformed FCC phase of copper at a range of piston velocity from 0.8 km/s to 3.0 km/s for three crystallographic direction of (**a**) 〈100〉, (**b**) 〈110〉 and (**c**) 〈111〉. The disordered phases (lattices with no definitive local crystal ordering as reported by adaptive-CNA)^32^ has not been presented here.

The shock Hugoniot plane (pressure and density) have been plotted for the above three simulation methods and three different orientations in Fig. 1. The results demonstrate close comparison with experimental shock data of poly-crystalline copper^37^. Figure 2 represents volume fraction of different lattice configuration at different shock intensities in different directions. Similar results have been obtained from NEMD and MSST simulation studies. Body-centered phases has been observed for shock loading along 〈100〉 and 〈110〉 directions but not along 〈111〉 direction. It should be mentioned that the a-CNA is an improvement over conventional CNA in which a fixed cutoff distance used. Conventional CNA has been replaced by variable cutoff to account for positional fluctuation as a result of high temperature or inhomogeneous atomistic strain distribution. However, it should be mentioned that even though this method gives an approximate percentage of volume fraction for different phases in the mix, it is unable to distinguish the symmetry in the crystal lattice structures (such as triclinic, monoclinic, orthorhombic, tetrahedral, etc.) apart from the cubic phase.

**Figure 3:**
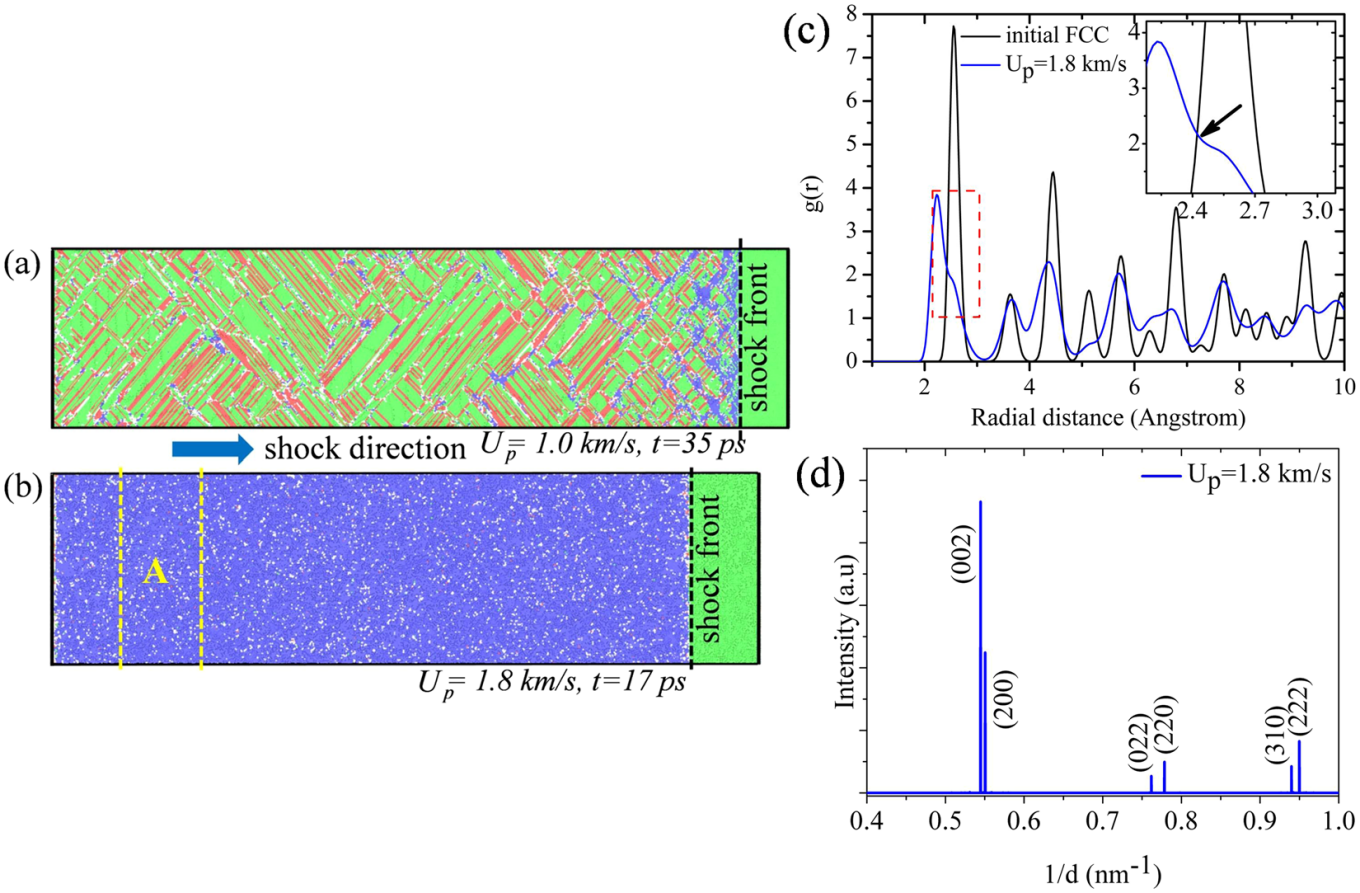
Shock-induced deformation matrix of Cu(100) has been represented for piston velocity of (**a**) 1.0 km/s and (**b**) 1.8 km/s. Local lattice environment has been analyzed through (**c**) radial distribution function (RDF) and (**d**) X-ray diffraction pattern. For identifying local lattice environment adaptive-common neighbor analysis (a-CNA)^32^ has been employed as depicted in (**a**) and (**b**), where, color green, blue, red and white represent FCC, BCC, HCP and unidentified phase respectively. Black dashed line indicates the position of shock front at particular time instance as mentioned in the figure. Region ‘A’ (a 150 Å thick slice far behind the shock front) as shown in (**b**) (bounded by yellow colored dashed line) has been considered for characterizing lattice structure through RDF and XRD, as represented in (**c**) and (**d**) respectively.

In Fig. 3(a) and (b), shock induced deformed matrix (obtained using NEMD simulations) is presented for Cu(100) for the piston velocities of 1.0 km/s and 1.8 km/s. Based on utilizing neighbor counting based local lattice structure identification method, adaptive-common neighbor analysis (CNA)^32^, it can be observed that for low-intensity shock compression (e.g. *U*_*p*_ = 1.0 km/s) dislocation based plasticity (resulting in the formation of HCP lattice due to the slip assisted change in stacking sequence of atomic layers) predominates. At piston velocity of 1.0 km/s, just behind the shock front a small portion of BCC lattice (blue colored atoms as depicted in Fig. 3(a)) is observed to be nucleated due to shock-induced uniaxial strain of around 5.4%; however as soon as the shock passes and moves further the observed BCC lattices reverts back to original FCC through post-shock relaxation of shear stress and associated strain. On the other hand, for higher piston velocities (e.g. *U*_*p*_ = 1.8 km/s resulting in shock pressure of ~105 GPa) as soon as the shock interacts with the target the entire sample transformed into body-centered phase (plastic strain along the shock propagation direction, is ~23.8%) of copper (see Fig. 3(b)) as obtained through adaptive-CNA analysis and these body centered phases are observed to survive post-shock relaxation upto 18.5 ps. RDF plots (see Fig. 3(c)) and XRD plots (see Fig. 3(d)) also demonstrate a clear signature of body-centered tetrahedral phase. More details regarding these two analysis methodologies have been presented in the next paragraphs.

**Figure 4:**
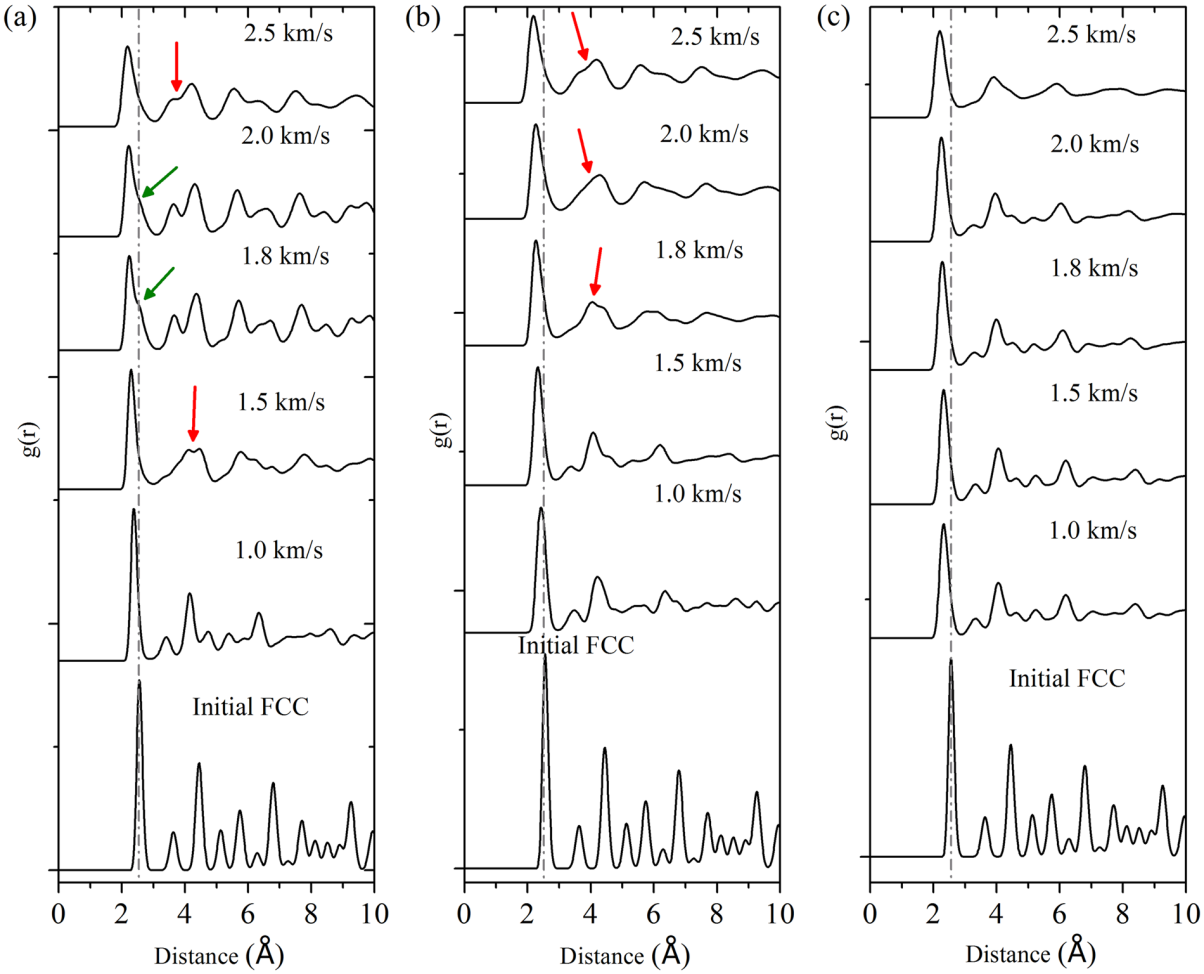
Calculated radial distribution function (RDF) of the deformed micro-structure shocked at a range of impact velocity of 1.0 km/s to 2.5 km/s, along the crystallographic direction (**a**) 〈100〉, (**b**) 〈110〉 and (**c**) 〈111〉. In this stacked plots of RDFs, the gray colored dash-dot line represents the shift of the position of the first neighbour with respect to initial undeformed sample. Green arrows indicate the observance of the ‘kink’ like shoulder inflamation region, whereas, red colored arrows point to the ‘wavy’ nature of second neighbour position.

It should be mentioned that RDF plots have been used to demonstrate structural phase transition in materials^38, 39^. Thus, RDF plots are also considered to analyze in this work. Figure 4 shows RDF of the atoms which are calculated from the trajectory of the shocked specimen up to 8 Å and averaged out over 5 ps time scale to obtain a clear picture regarding the temporal dependency as well as probability to obtain the 1st, 2nd and 3rd neighbors inside the specimen under the shocking situation. Results shown in Fig. 4, correspond to EAM based MSST simulation results. Similar results have also been obtained for NEMD simulations and have been found to be identical. Typically a body-centered phase has certain typical signatures such as the presence of ‘shoulder region’ in the descending part of the first coordination shell which indicates the presence of two merged peaks. The peaks refer to the probabilities of atom neighbors near one another. In several situations, a combination of thermal broadening of the peaks and the ‘shoulder region’ results in the formation of ‘kink’ regions (or regions with two different slopes) in the descending part of the first coordination shell. These signatures of body-centered phase (typically the presence of the ‘kinks’) are observed from the RDF profiles corresponding to 〈100〉 shock loading direction for piston velocities of 1.8 to 2.0 km/s. The corresponding temperature and pressure range of these piston speeds are ~1788–2286 K and ~90–105 GPa, respectively. Another typical signature of the possibility of the presence of body-centered phase is merging of second and third coordination peaks resulting in a ‘wavy’ nature of the RDF curve. This feature could be observed for piston velocity of 1.5 km/s (in the 〈100〉 shock loading direction) which does not show any clear peak for second and third coordination shell, rather the curve at this region of 2–4 Å is ‘wavy’ in nature. Similar ‘wavy’ nature of the second and third coordination shell peaks could also be observed for 2.5 km/s piston velocity for the 〈100〉 shock loading direction and for piston velocities of 1.8 to 2.5 km/s for the 〈110〉 shock loading direction. Apart from that, a general characteristics such as a leftward shift of first coordination shell peak, thermal broadening, and decrease in intensity of the peak as well as the gradual loss of coordination (typified by flattening nature of the higher coordination shell peaks with increase in piston velocities) could be observed for shock loading in both the directions. No specific ‘kink’ and/or ‘wavy’ nature could be observed for the shocks along 〈111〉 direction, signifying almost no probability of transition to a body-centered phase. It should also be noted that at this point, even though there are presence of certain signatures for body-centered phase from the RDF plots for the shocks along 〈100〉 and 〈110〉 directions, but these RDF profiles are not able to comment on whether the body-centered phase is cubic, tetrahedral, orthorhombic or some other lattice structures. It may also happen that due to the presence of low volume fraction of the body-centered phase the effect/signature has not been reflected in the RDF plots.

**Figure 5:**
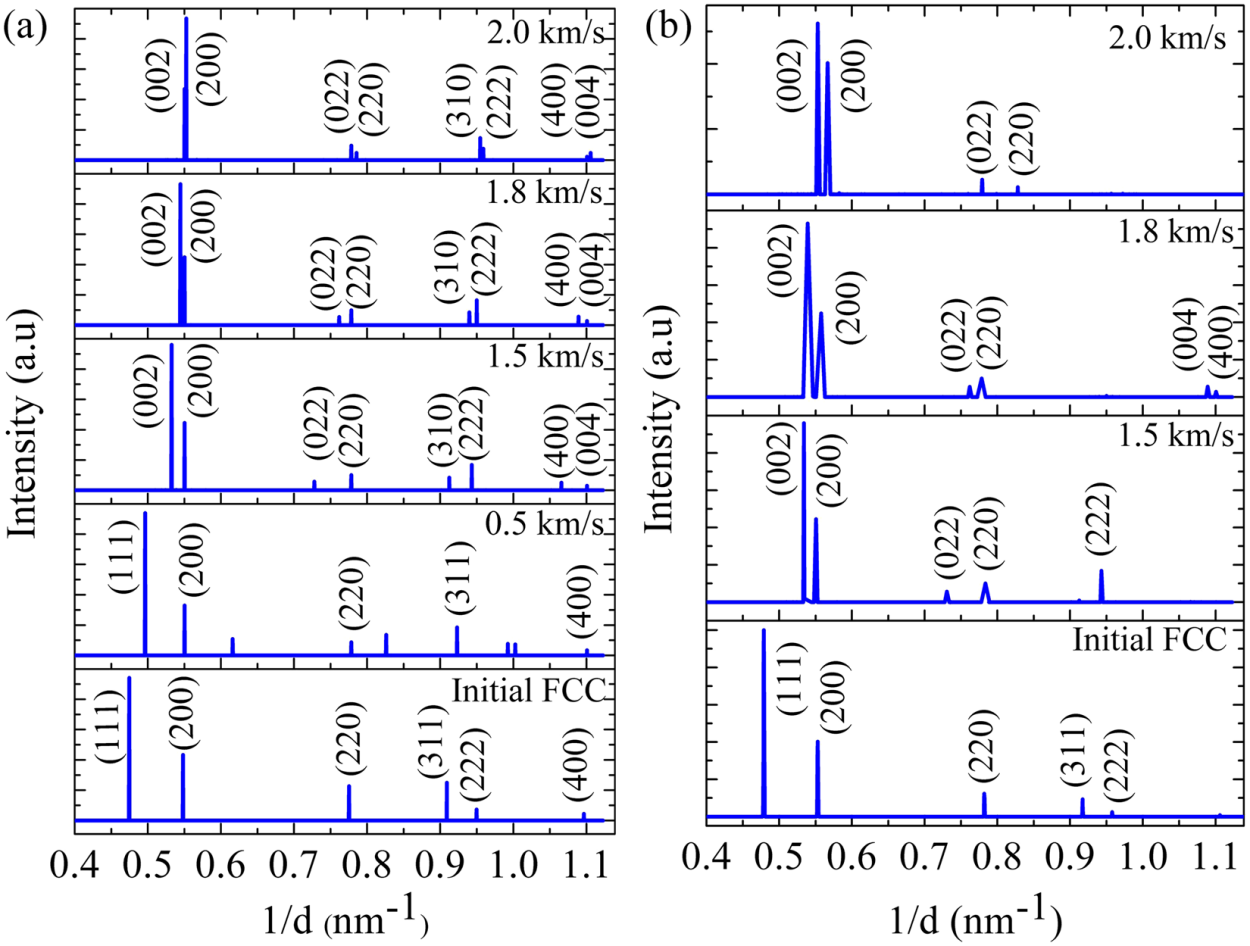
X-ray diffraction patterns Cu along 〈100〉 crystallographic direction at ambient temperature and pressure as well as shock compressed conditions at different piston velocities demonstrating structural phase transition: (**a**) Results obtained from classical MD simulations (which includes both NEMD and MSST simulations) (**b**) Results obtained from *ab* − *initio* MD simulations.

To get deeper insights, x-ray diffraction patterns have been simulated from the atomistic trajectory data. At ambient temperature and pressure conditions, XRD plots (refer Fig. 5(a)) for Cu shows primary peak corresponding to (111) plane at a interplanar spacing (*d*_*hkl*_) of 2.107 nm (i.e. 2*θ* value of 42.92°, where lattice parameter a = 3.615 Å; volume/atom = 11.68 Å^3^, is in good agreement with ICDD copper file No. 040836). The initial structure is an FCC since h, k, l shows either all odd or all even values^40^. Lattice deformation at piston velocity of 0.5 km/s in Cu(100) is demonstrated through a slight shift in the interplanar spacing of the (111) plane to 2.014 nm (i.e. 2*θ* of 45.05°). Amongst the nine peaks for the piston velocity of 0.5 km/s, five depict FCC phase with a = 3.512 Å and others (peaks 3, 5, 7, 8) illustrate the HCP phase, nucleated as a result of shock-induced plastic deformation. At piston velocities of 1.5 km/s and above we have observed even values of (h + k + l) thereby demonstrating a body-centered lattice structure^40^. For 1.5, 1.8, 2.0, and 2.5 km/s piston velocity the (111) peak is no longer observed and the (200) peak splits into two parts corresponding to (200) and (002), demonstrating a typical characteristic of tetragonal lattice formation. It should be pointed out that (002) plane spacing in a tetragonal lattice structure differs from the other two planes ((200) and (020)); whereas all the three planes have same spacing for a cubic structure^40^. Consistent with the (200) peak split higher order peak splits has also been observed for MD simulations. XRD analysis of *ab* − *initio* MD simulation results (shown in Fig. 5(b)) demonstrate a rigorous proof of shock-induced structural phase transition from FCC to BCT lattice structure (characterized by splitting of the peak at (200) into (002) and (200)) at piston velocities 1.5 km/s and above till 2.5 km/s for shock compressed Cu in the 〈100〉 direction. Peak broadening along with an increase in temperature of the samples (associated with higher piston velocities) is also observed in XRD analysis of *ab* − *initio* MD simulations which are not prominent in the MD simulation results. Results obtained from the classical MD simulations shows (200) peak for initial FCC structure at the interplanar spacing of 1.824 nm (2*θ* = 49.95°); (002) and (200) peaks at the interplanar spacing of 1.877 and 1.817 nm (i.e. 2*θ* = 48.4° and 50.1°), respectively for 1.5 km/s piston velocity; (002) and (200) planes at the interplanar spacing of 1.836 and 1.817 nm (2*θ* = 49.73° and 50.38°), respectively for the piston velocity of 1.8 km/s; (002) and (200) planes at the interplanar spacing of 1.817 and 1.809 nm (2*θ* = 49.95° and 50.38°), respectively for 2.0 km/s piston velocity. The x-ray diffraction profiles obtained from the atomistic trajectory of *ab* − *initio* MD (AIMD) simulations show (200) and (002) peaks are at the interplanar spacing of 1.873 and 1.817 nm (2*θ* = 48.61° and 50.37°) respectively for piston velocity of 1.5 km/s; (200) and (002) planes at the interplanar spacing of 1.854 and 1.794 nm (2*θ* = 49.39° and 50.86°), respectively for 1.8 km/s piston velocity; (200) and (002) planes at the interplanar spacing of 1.809 and 1.765 nm (2*θ* = 50.57° and 51.94°), respectively for 2.0 km/s piston velocity. Thus the x-ray peak features, including the relative intensity of the peaks, obtained from EAM-based classical MD simulation and AIMD simulations are in good agreement with each other, which establish the capability and accuracy of the EAM-Mishin potential of copper at shock-loaded state.

**Figure 6:**
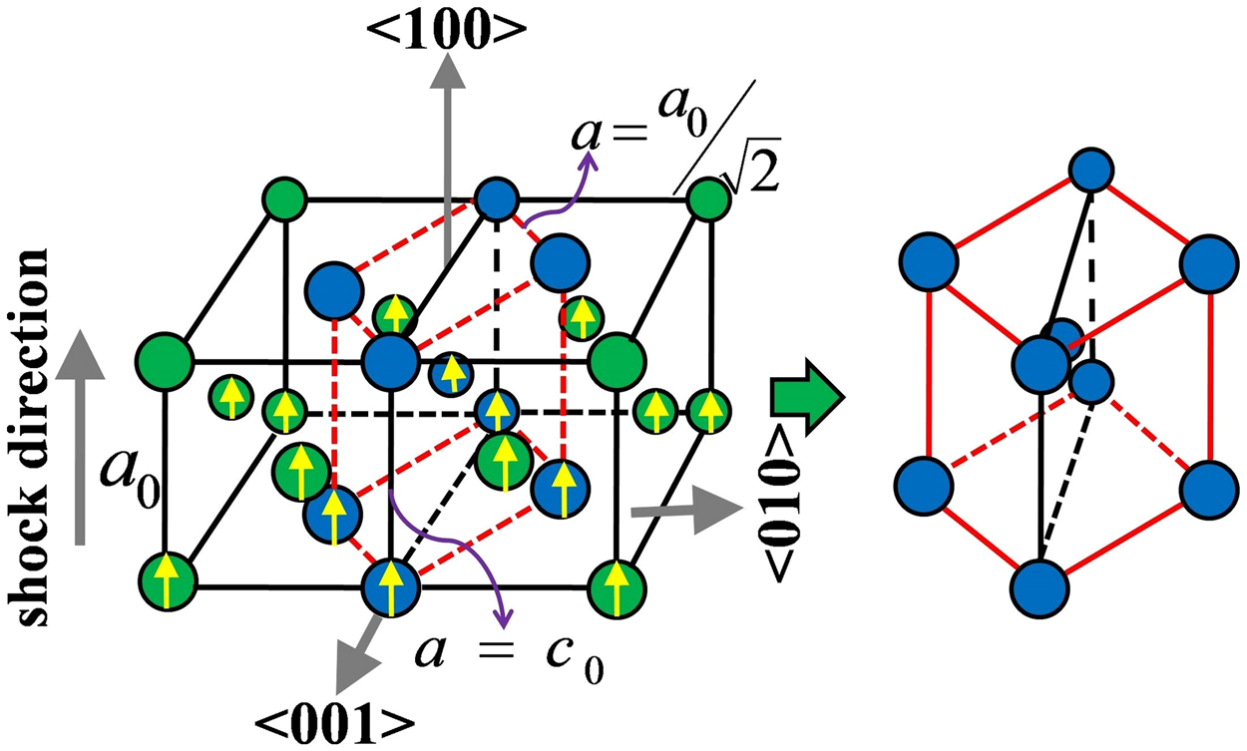
Schematic of atomistic mechanism of structural phase transition in Cu(100) direction. BCT unit cell in between two FCC lattice has been shown by blue colored balls connected by red dashed lines. Yellow colored arrow signifies uniaxial atomic displacement under shock load.

The mechanism as observed in this structural phase transformation, FCC-to-BCT, in Cu(100) is primarily an uniaxial lattice compression accompanied by lattice rotation through finite volume change. As shown in the schematic diagram (see Fig. 6), a lower symmetry tetragonal body-centered unit cell (with $\frac{c}{a}=\frac{1}{\sqrt{2}}$) can be assumed (connected via red dashed line in Fig. 6) within the two consecutive high symmetry FCC lattice unit cells. As a consequence, adequate lattice compression of $\frac{1}{\sqrt{2}}$, i.e. strain of ~30% along 〈100〉 crystallographic direction transforms this low symmetry BCT unit cell to BCC lattice. However, at a piston velocity of 1.5 km/s (i.e. shock pressure and temperature of ~80 GPa and ~1130 K, respectively) spatially averaged uniaxial lattice strain is obtained as ~21.75%, which is insufficient for complete transformation to BCC and eventually a body-centered phase with tetragonal symmetry (tetragonality is ~3.3%, $\frac{c}{a}=0.967$) is identified. Successive increment of the applied strain by employing higher intensity shock, e.g. piston velocity of 1.8 km/s (shock pressure and temperature of ~100 GPa and 1520 K, respectively with spatially averaged uniaxial lattice strain is obtained as ~23%), the displacement of the atoms to attain higher symmetry BCC lattice is facilitated and that is why the measured tetragonality of the obtained BCT phase is decreased to ~1%. At 2.0 km/s shock speed, further reduction of tetragonality can be obtained with the increase in spatially averaged uniaxial lattice strain to ~25%. At the much higher intensity of shock, beyond piston velocity of 2.0 km/s, amorphous solid phase along with several small crystallites (with short range ordering up to ~10 Å) has been observed to exist prior to melting.

**Figure 7:**
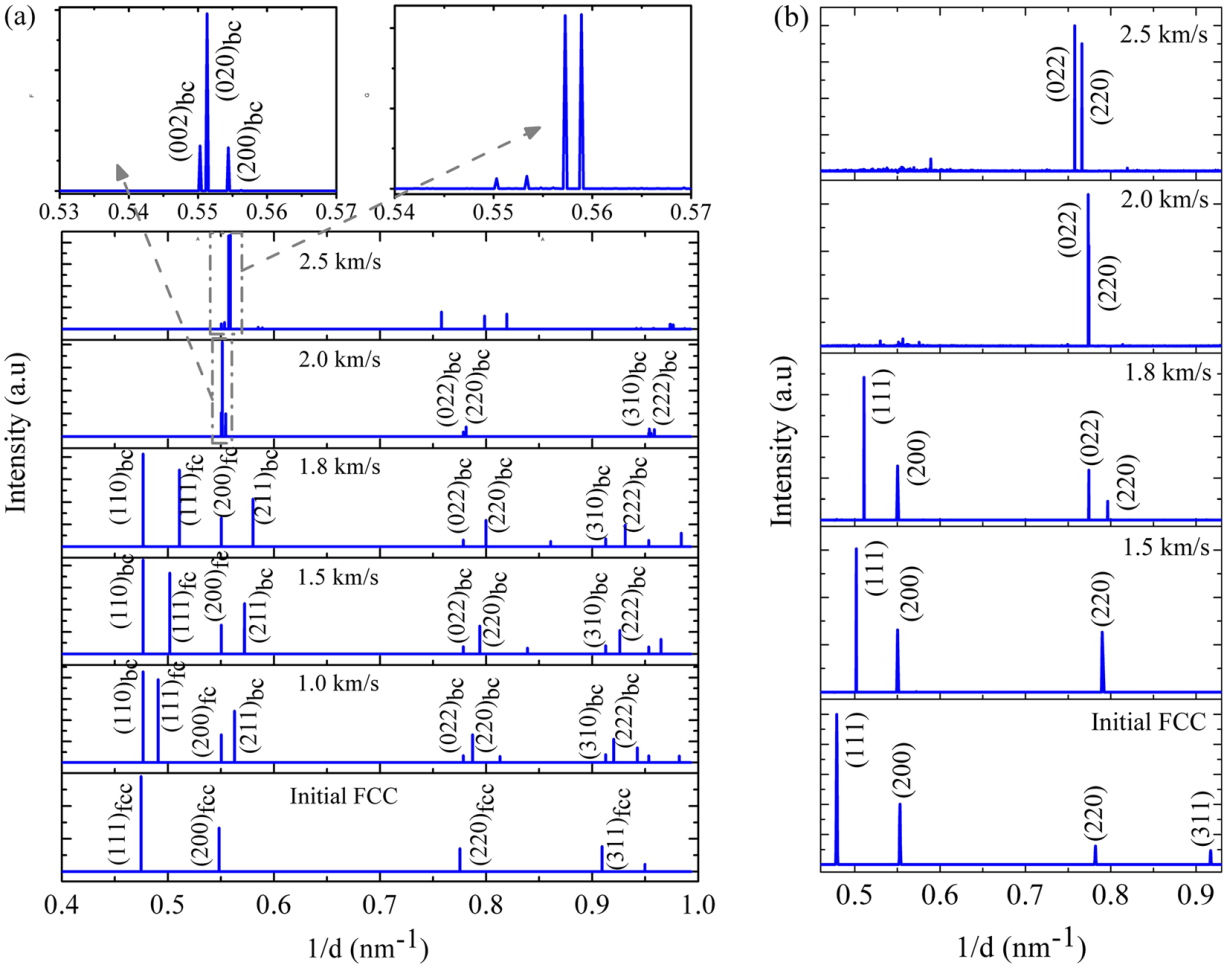
X-ray diffraction patterns of Cu along 〈110〉 crystallographic direction at ambient temperature and pressure as well as shock compressed conditions at different piston velocities demonstrating structural phase transition: (**a**) Results obtained from classical MD simulations (which includes both NEMD and MSST simulations) (**b**) Results obtained from *ab* − *initio* MD simulations.

Signatures of body-centered phases (even values of (h + k + l)) has also been observed (see Fig. 7(a)) for the shocks along 〈110〉, at a piston velocity of and above 1.0 km/s. However, the nucleated body-centered phase has been observed to co-exist with the parent FCC matrix at the piston velocities of 1.0–1.8 km/s. From the x-ray diffraction profile as obtained from the EAM based MD simulations, at the piston velocity of 2.0 km/s the (200) plane has been observed to split into (002), (020) and (200) (with the interplanar spacing of 1.817, 1.814, and 1.804 nm, respectively, i.e. 2*θ* = 50.23, 50.33, and 50.63°, respectively) thereby demonstrating the formation of orthorhombic lattice^40^ (as earlier postulated by Hirth *et al*.^27^). At 2.5 km/s, four peaks can be observed near to a 2*θ* angle of 50°, i.e. at the range of the interplanar spacing of 1.818–1.786 nm, which exhibits an unknown/unidentified crystal lattice structure. The *ab* − *initio* molecular dynamics (AIMD) based MSST simulations (as shown in Fig. 7(b)) however illustrate only splitting of the (220) planes into (022) and (220) plane at piston velocities 1.8 km/s and above. Even values of (h + k + l) for the x-ray peaks obtained for piston velocities of 2.0 km/s and above, indicates towards the formation of a body-centered lattice as well. Even though peak splitting is observed for the (220) planes, unlike the primary (200) plane for the shocks at the piston velocity of 2.0 km/s and above. This feature thereby evidences that a body-centered phase is being formed under shock-loading, but the exact crystal lattice structure (i.e. whether tetrahedral or orthorhombic) cannot be ascertained easily which is thus open for further investigation. In the plots (refer Fig. 7(a)) obtained from MD simulations, ‘bc’ and ‘fc’ indicates planes corresponding to body-centered and face-centered lattice respectively. The two boxes indicated by a gray colored arrow represents the zoomed view near the (002) plane for the impact velocity of 2.0 and 2.5 km/s. For 2.0 km/s piston velocity (002), (020) and (200) planes have been observed with the peaks at the interplanar spacing of 1.817, 1.814, and 1.804 nm, respectively. X-ray diffraction patterns as obtained from *ab* − *initio* MD simulations show (022) and (220) peaks are at the interplanar spacing of 1.292 and 1.256 nm (2*θ* = 73.71° and 76.20°), respectively for piston velocity of 1.8 km/s; (022) and (220) peaks are at the interplanar spacing of 1.292 and 1.290 nm (2*θ* = 73.28° and 74.45°), respectively for piston velocity of 2.0 km/s; (022) and (220) peaks are at the interplanar spacing of 1.320 and 1.305 nm (2*θ* = 71.66° and 72.40°), respectively for piston velocity of 2.5 km/s.

In conclusion, while performing shock simulation of Cu using different shock-intensities and different orientations of the lattices, we have stumbled upon a discovery of the existence of the body-centered phase of Cu, shock compressed along 〈100〉 direction. Using three different local lattice structure identification methods, these results have been derived based on classical MD simulations considering well accepted EAM-Mishin potential for Cu. These results have also been validated through computationally expensive *ab* − *initio* MD simulations at certain piston velocities. An extensive density functional theory (DFT) study could be undertaken to determine the dip in the energy associated with the phase transition in Cu under shock loading of different intensities and in different directions which will be addressed by the authors in some later publications. Laser ablated shock compression experiments with the facilities for *in*-*situ* real-time XRD diffraction analysis, can also validate this numerical study of the shock-induced phase transformation in Cu made in this article.

## Methods

Detailed molecular dynamic simulations (NEMD and MSST)^33^ have been carried out in this work in which single crystal Cu in different orientations has been subjected to shock loads of varying intensities (piston velocity from 0.8 to 3.0 km/s). The force potential used for Cu in MD simulations is the many-body EAM potential developed by Mishin *et al*.^31^. It should be noted that EAM-Mishin potential for Cu has been parameterized based on *ab* − *initio* and tight-binding methods and is well accepted by the research community as the standard for simulations of Cu both at ambient temperature and pressure conditions and also at high pressure and temperature conditions. The potential shows good agreement with experimental elastic constants, experimental phonon dispersion curves thereby demonstrating its global reliability. Apart from the calculation of lattice properties, the potential has also been extensively tested for various structural energies and transformation paths. It should be noted that EAM potential is well referenced in many papers on shock loading of Cu^41–43^). Single crystal copper with periodic boundary condition in all orthogonal directions, is created with various crystal orientations, [100], [110] and [111] for the MD simulations. For details of the samples, please see Tables 1 and 2 for EAM based NEMD and MSST simulations.

**Table 1:** Necessary details of the initial configurations of the samples for NEMD simulations with desired orientations.

Sample no.	No. of crystal lattice units	Orientation			Size (nm)			No. of atoms
		X	Y	Z	Shock dirn. X	Y	Z	
1.	500 × 100 × 100	〈100〉	〈010〉	〈001〉	180.75	36.15	36.15	20000 × 10^3^
2.	350 × 80 × 70	〈110〉	〈001〉	〈1$\bar{1}$0〉	178.93	28.92	35.79	15680 × 10^3^
3.	280 × 60 × 60	〈111〉	〈1$\bar{1}$0〉	〈11$\bar{2}$〉	175.32	30.67	35.42	16128 × 10^3^

The mass density of all the samples are 8.806 g/cm^3^ at ambient temperature and pressure.

**Table 2:** Necessary details of the initial configurations of the samples with desired orientations used for EAM MD simulations.

Sample no.	No. of crystal lattice units	Orientation			Size (Å)			No. of atoms (× 10^3^)
		X	Y	Z	X	Y	Z	
1.	20 × 20 × 20	〈100〉	〈010〉	〈001〉	72.30	72.30	72.30	32
2.	15 × 20 × 15	〈110〉	〈001〉	〈1$\bar{1}$0〉	77.708	73.023	77.7082	36
3.	12 × 15 × 12	〈111〉	〈1$\bar{1}$0〉	〈11$\bar{2}$〉	76.389	77.708	72.012	34.56

The mass density of all the samples are 8.806 g/cm^3^ at ambient temperature and pressure.

Three different analysis methods have been used to identify the shock equilibrated crystal structure of the samples - adaptive Common Neighbor analysis (a-CNA)^32^ (lattice structure identification in multiphase systems based on coordination number of atoms), Radial distribution function (RDF - which calculate the probability of finding out two neighbors at a certain distance apart) analysis and X-ray diffraction (XRD) analysis. Identification by any one method is not reliable since it may provide a misleading prediction, as has been discussed in the article.

Computationally expensive *ab* − *initio* molecular dynamics (AIMD) simulations (using AM05 exchange-correlation function) has been carried out (simulation details and sample sizes have been presented in Table 3) and through x-ray diffraction analysis the presence of the new phase is demonstrated.

**Table 3:** Necessary details of the initial configurations of the samples with desired orientations used for *ab* − *initio* molecular dynamics (AIMD).

Sample no.	No. of crystal lattice units	Orientation			Size (Å)			No. of atoms
		X	Y	Z	X	Y	Z	
1.	4 × 4 × 4	〈100〉	〈010〉	〈001〉	14.46	14.46	14.46	256
2.	2 × 4 × 2	〈110〉	〈001〉	〈1$\bar{1}$0〉	10.224	14.46	10.224	128
3.	2 × 2 × 2	〈111〉	〈1$\bar{1}$0〉	〈11$\bar{2}$〉	12.52	10.23	11.81	128

The mass density of all the samples are 8.814 g/cm^3^ at ambient temperature and pressure.

In the NEMD simulations, the initial configurations of pure single crystal with desired orientations have been created and equilibrated by applying isothermal-isobaric, NPT ensemble integration scheme (for 100 ps with timestep size of 1 fs) along with Nose-Hoover thermostat algorithm at ambient temperature and pressure condition. Periodic boundary conditions (PBC) are maintained in all three orthogonal directions during equilibration period. The details of the samples, including, crystallographic orientation, sample dimensions and the total number of atoms, has been given in the Table 1. Please note, special care has been taken while choosing the simulation domain size for ensuring elimination of any artifact of transverse periodic boundary^44, 45^ over the shock-induced plasticity mechanisms. The simulation domain size for this study has been chosen in such a way that it is adequate to contain the entire shock in transverse directions and also the shocks are steady in nature in each simulation with rise time of ~0.51–1.1 ps for the range of piston velocities of 3.0–1.0 km/s respectively for all above-mentioned crystal orientations. To generate a unidirectional planar shock wave in direct-shock/NEMD simulations, so-called standard ‘momentum-mirror’ method is applied to the equilibrated initial configurations conjointly with the micro-canonical ensemble, NVE, to achieve the conservation of energy during shock propagation. The size of the timestep during all shock simulation is 1 fs unless otherwise mentioned. All MD simulations have been accomplished by LAMMPS^46^ open-source package.

In order to carry out simulations with larger timescales, MSST algorithm (multiscale shock technique^47^ which is implemented under the LAMMPS^46^ framework and has been tested for simulating a unidirectional planar shock propagation for various class of materials^48–51^ with sufficient accuracy) has been used to perform simulations up to 10 ns after the passage of shock. The necessary sample details have been given in the Table 2. MSST is a simulation technique based on the Navier–Stokes equations for compressible flow and follows a Lagrangian point through the shock wave which is accomplished by time evolving equations of motion for the atoms, as well as volume of the computational cell to constrain the stress in the shock propagation direction to the Rayleigh line and the energy of the system to the Hugoniot energy condition. While performing the MSST simulations, care has been taken to ensure that there is no significant drift in energy at any time instance with the chosen values of ‘q’ and ‘tscale’ (parameters required in MSST simulation) for a particular shock intensity. Although, it should be noted here that any choice of ‘q’ (chosen as 1 for our simulations) and ‘tscale’ does not affect the essential generality of the background physics i.e. typically does not affect the obtained results from the simulations.

Since *ab* − *initio* calculations are computationally expensive compared to MD simulations, thereby a comparatively smaller number of atoms are taken to simulate the system. It should be noted that the essential generality of the physics remains unaltered in the simulation with a limited number of atoms as used in *ab* − *initio* molecular dynamics (AIMD). The necessary sample details have been given in the Table 3. During equilibration of the samples with desired lattice orientation (a 3D periodic box), we use a time-step of 1.0 fs and the Nose-Hoover thermostat for NVT ensemble calculations at 300 K with exchange-correlation functional of Armiento–Mattsson^52, 53^, which describes crystalline solids with better accuracy^54^ than the other functional of the same class (the density and gradient based functionals local density approximation (LDA)^55^, PBE^56^, BLYP^57, 58^ and RPBE^59^). The same MSST algorithm^33^ has been utilized for shock compression through AIMD simulations. All the AIMD simulations are up to 15–20 ps in timescale and an averaged energy drift from the Hugoniot energy condition has been observed as less than ~1%. Spin-resolved Armiento-Mattsson exchange-correlation function^52, 53^ (which includes both density gradients similar to that of GGA (Generalised gradient based approximation) based methods and surface specific estimates for both exchange and correlation energies) has been used for this simulations. It should be noted that the above-mentioned functional have shown significant fidelity for different materials such as semiconductors, simple metals, transition metals, alkalihalides and oxides^60, 61^. In these simulations, a plane wave cutoff of 250 Rydberg has been estimated as sufficient to get tight convergence in stress tensor. A wave function convergence criterion of 10^−6^ a.u is used for all simulations until otherwise mentioned. These AIMD simulations are accomplished by the program QUICKSTEP^62^ which is implemented under the program suit of CP2K^63^ open source software package.

To calculate the x-ray diffraction pattern of the shocked samples from the atomistic trajectories, the diffraction algorithm^64^ (as implemented in LAMMPS)^46^ has been utilized in which a 3D mesh of reciprocal lattice points is built on a rectangular grid with certain reciprocal space mesh size. The diffraction intensity at each reciprocal lattice point has been computed by performing spherical integration and corresponding normalization of intensity data by assuming all orientations are equally probably. During the calculation of diffraction intensity of all the samples shocked at different piston velocities, the 3D reciprocal grid spacing has been chosen as c1/X, c2/Y, and c3/Z; X, Y, and Z are the dimensions of the simulation box. It should be noted that since this is a simulation study of the x-ray diffraction pattern, the calculated diffraction profiles has been presented as a function of the inverse of interplanar spacing (d _*hkl*_). The wavelength has been chosen as 1.541838 nm. The use of inverse spacing of the simulation dimensions helps to ensure that points examined within the reciprocal space mesh may be close to the point of Bragg reflection. Series of calculations with the values of c1,c2,c3 as {0.0625,0.0625,0.0625}, {0.125,0.125,0.125}, {0.5,0.5,0.5}, {1,1,1}, {2,2,2} and {3,3,3} were done, but the computed diffraction patterns didn’t showed significant dependency of the mesh resolution over the diffraction pattern. All the results shown in this paper were computed by using 1, 1 and 1 as c1, c2, and c3 respectively. This {1,1,1} values were evaluated as the optimized and accurate for all shock intensities. To examine the effect of finite volume over the simulated x-ray diffraction profiles, we have performed NEMD shock simulation taking larger transverse Y and Z direction (the simulation box dimension is 180.75 × 72.3 × 72.3 nm^3^) and have calculated diffraction pattern (see the supplementary document); no significant differences in diffraction peak characteristics has been observed. To investigate the dynamic evolution of the BCT phase over the simulated time span, x-ray diffraction pattern has also been calculated for various time instances of the atomistic trajectory and no significant differences could be observed in peak profiles (see the supplementary document), which indicates the stability of the BCT phase up to 10 ns timescale.

## Electronic supplementary material

Supplementary information
