# Supplementary material for: A metastable phase of shocked bulk single crystal copper: an atomistic simulation study
Source: Sci Rep. 2017 Aug 4;7:7337. doi: 10.1038/s41598-017-07809-1 (PMC5544681; doi:10.1038/s41598-017-07809-1)
Supplement: Supplementary file 1 — Supplementary information [file 41598_2017_7809_MOESM1_ESM.pdf]

A metastable phase of shocked bulk single crystal copper: an atomistic simulation study

Anupam Neogi*
Advanced Technology Development Center, Indian Institute of Technology Kharagpur, Kharagpur 721302, India

Nilanjan Mitra†
Center for Theoretical Studies, Indian Institute of Technology Kharagpur, Kharagpur 721302, India
(Dated: June 2, 2017)

To examine the effect of finite volume over the simulated x-ray diffraction profiles, we have performed NEMD shock simulation taking larger transverse Y and Z direction (the simulation box dimension is 180.75× 72.3× 72.3 nm^3) and have calculated diffraction pattern (see Fig. 1); no significant differences in diffraction peak characteristics has been observed. To investigate the dynamic evolution of the BCT phase over the simulated time span, x-ray diffraction pattern has also been calculated for various time instances of the atomistic trajectory and no significant differences could be observed in peak profiles (see Fig. 2), which indicates the stability of the BCT phase up to 10 ns timescale.

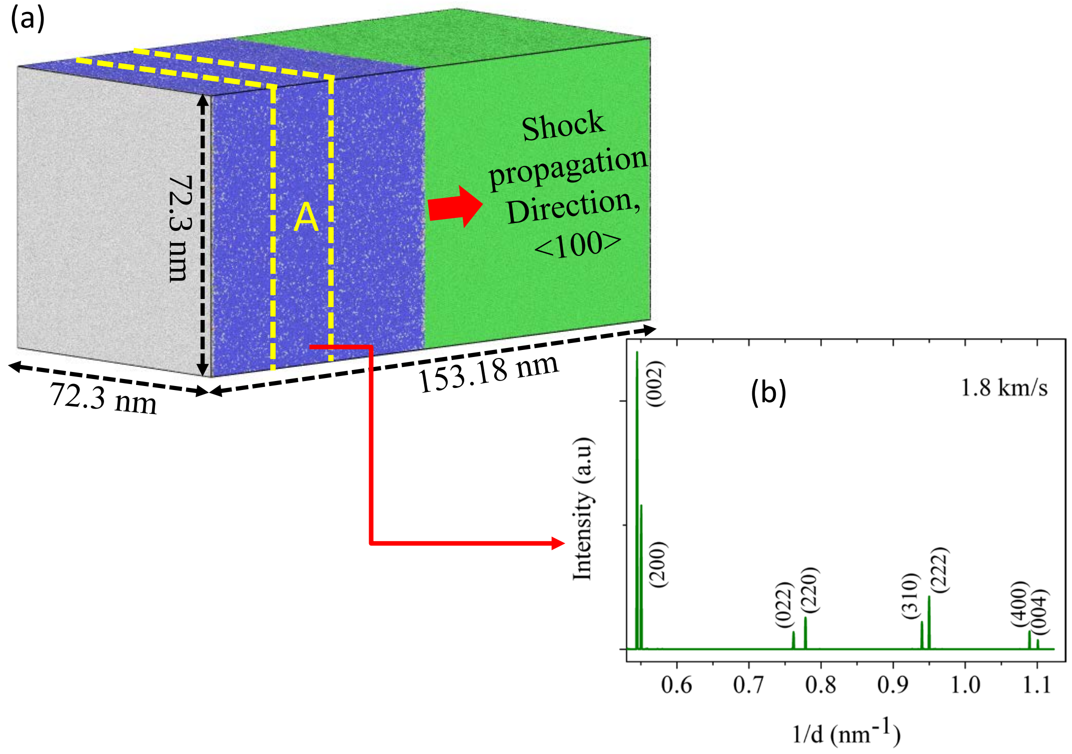

FIG. 1: (a) The deformed microstructure (as obtained by adaptive-CNA analysis) of shocked Cu(100) at a piston velocity of 1.8 km/s. Blue, Green, and white color indicates body-centered, face-centered, and unidentified phases by a-CNA method. The initial simulation box dimension is 180.75× 72.3× 72.3 nm^3. (b) simulated x-ray diffraction pattern of the region, 'A' (width of 300 Å).

* Electronic address: anupamneogi@gmail.com
† Electronic address: nilanjan@civil.iitkgp.ernet.in

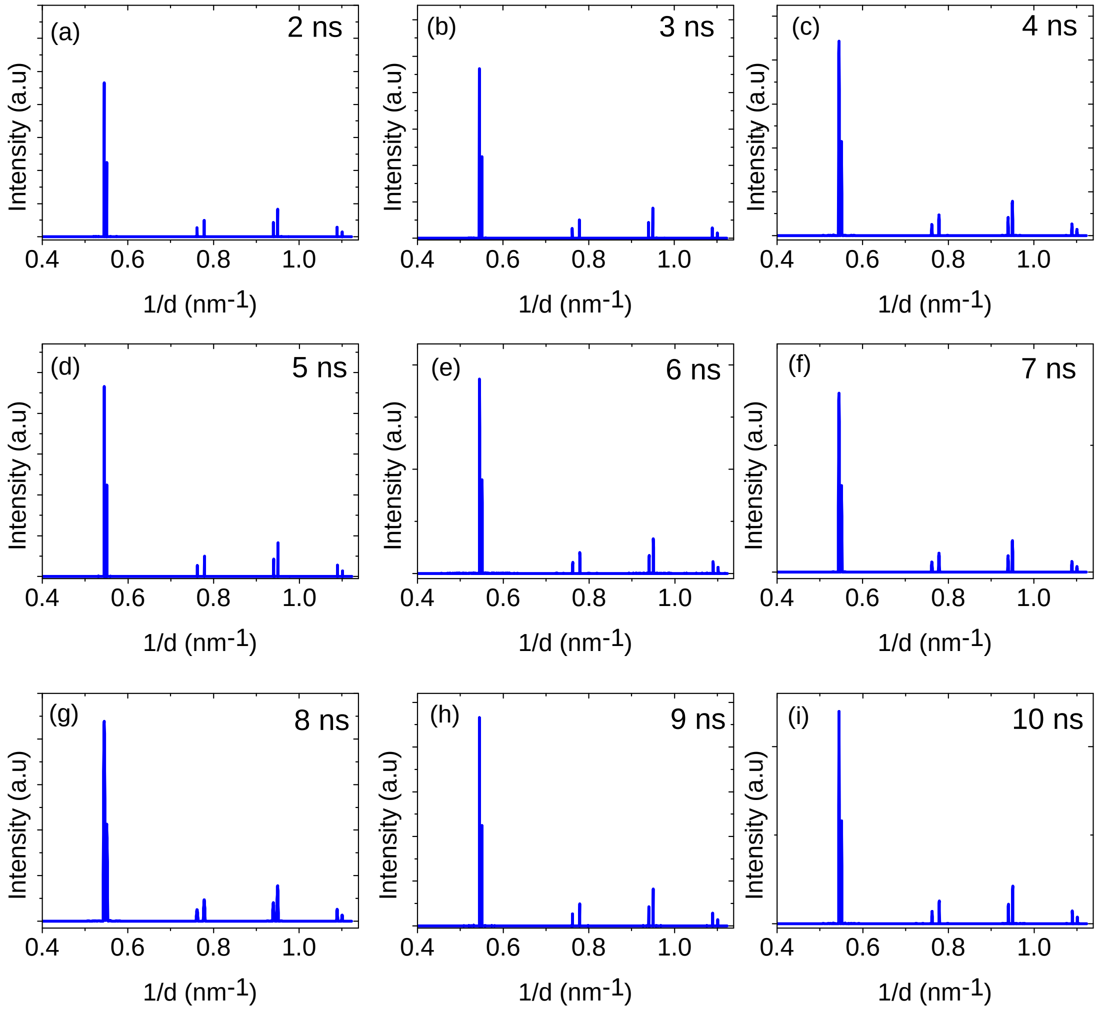

FIG. 2: X-ray diffraction pattern of shocked copper (100) at different time instances for a piston velocity of 1.8 km/s.
